# RE: The Rumpelstiltskin effect: therapeutic repercussions of clinical diagnosis

**DOI:** 10.1192/bjb.2025.10170

**Published:** 2025-12

**Authors:** Ronald W. Pies

**Affiliations:** MD, Professor Emeritus of Psychiatry, Department of Psychiatry, SUNY Upstate Medical University, Syracuse, NY, USA

Kudos to Prof. Levinovitz and Dr Aftab^1^ for a rich and important conceptual piece on ‘diagnosis’ and naming. In response, I offer the following comments.

First, the act of ‘naming’ something – or changing a name – has mythic and spiritual resonances going back to Biblical times; e.g. when, after he wrestles with an angel, Jacob’s name is changed to Israel, meaning ‘One who struggles with God’. Another example is Saul’s name change to ‘Paul’ in the New Testament. Although a ‘diagnosis’ certainly does have a ‘naming’ function, the etymology of the term points to its epistemic content (*gnosis*) and value; literally, the term means ‘knowing the difference between’ (*dia*: across or between; *gnosis*: knowledge or knowing). So when, after a careful evaluation, the physician provides a ‘dia-gnosis’, he or she is both naming something and also asserting: ‘I have determined the difference between your condition and many, many others that you do not have’. And, in this area, a conversation with the patient may begin as regards the nature and significance of the diagnosis and its referent (e.g. rheumatoid arthritis, schizophrenia or whatever).

As a psychiatrist, I have found that a carefully obtained diagnosis – when presented tactfully and tentatively, and with adequate information – can indeed be affirming, validating and relieving to the patient. I have treated many patients whose mood swings and erratic behaviour went undiagnosed for many years – until, finally, bipolar disorder was ‘named’, diagnosed and successfully treated. Just hearing the name ‘bipolar disorder’ can be a source of relief and epistemic value for the patient, whose symptoms and dysfunction are no longer attributed to lack of self-discipline, ‘stress’, failure of self-understanding, etc.

Yes, a diagnosis – more accurately, the name of a diagnosis – can be unsettling and anxiety-provoking, especially if it carries pejorative connotations. In psychiatry, the term ‘borderline’ (as in borderline personality disorder) is one example. And if a diagnosis is presented to the patient as a fixed and essential feature of his or her personhood, damage may be done. The careful clinician takes pains to avoid such a (mis)presentation by emphasising that the person in treatment is not exhaustively defined by his or her diagnosis, that there are aspects of the condition over which he or she can exercise control, and that nothing is ‘written in stone’. Many patients recover from their illnesses, and most can be treated effectively.

In my view, there is a curious cultural–linguistic bias that often attaches to psychiatric diagnoses; i.e. they are often referred to as ‘labels’. We do not find this term used in general medicine – nobody says ‘I was labelled with cancer’ – or even in neurology, where many conditions do not have a well-characterised pathophysiology or aetiology. For example, nobody speaks of being ‘labelled’ with migraine headaches or idiopathic facial pain. My sense is that this disparity represents a form of anti-psychiatric prejudice.
